# A universal vaccine candidate against *Plasmodium vivax* malaria confers protective immunity against the three *Pv*CSP alleles

**DOI:** 10.1038/s41598-021-96986-1

**Published:** 2021-09-09

**Authors:** Alba Marina Gimenez, Ahmed M. Salman, Rodolfo F. Marques, César López-Camacho, Kate Harrison, Young Chan Kim, Chris J. Janse, Irene S. Soares, Arturo Reyes-Sandoval

**Affiliations:** 1 Nuffield Department of Medicine, The Jenner Institute, University of Oxford, The Henry Wellcome Building for Molecular Physiology, Roosevelt Drive, Oxford, OX3 7BN UK; 2 Department of Clinical and Toxicological Analyses, School of Pharmaceutical Sciences, University of São Paulo, São Paulo, SP Brazil; 3 Department of Parasitology, Leiden Malaria Research Group, Center of Infectious Diseases, Leiden University Medical Center, (LUMC, L4-Q), Albinusdreef 2, 2333 ZA Leiden, The Netherlands; 4 Instituto Politécnico Nacional, IPN, Av. Luis Enrique Erro S/N. Unidad Adolfo López Mateos, Zacatenco, CP 07738 Mexico City, Mexico

**Keywords:** Protein vaccines, Parasitology

## Abstract

Malaria is a highly prevalent parasitic disease in regions with tropical and subtropical climates worldwide. Among the species of *Plasmodium* causing human malaria, *P. vivax* is the second most prevalent and the most geographically widespread species. A major target of a pre-erythrocytic vaccine is the *P. vivax* circumsporozoite protein (*Pv*CSP). In previous studies, we fused two recombinant proteins representing three allelic variants of *Pv*CSP (VK210, VK247 and *P. vivax*-like) to the mumps virus nucleocapsid protein to enhance immune responses against *Pv*CSP. The objective of the present study was to evaluate the protective efficacy of these recombinants in mice challenged with transgenic *P. berghei* parasites expressing *Pv*CSP allelic variants. Formulations containing Poly (I:C) or Montanide ISA720 as adjuvants elicited high and long-lasting IgG antibody titers specific to each *Pv*CSP allelic variant. Immunized mice were challenged with two existing chimeric *P. berghei* parasite lines expressing *Pv*CSP-VK210 and *Pv*CSP-VK247. We also developed a novel chimeric line expressing the third allelic variant, *Pv*CSP-*P. vivax*-like, as a new murine immunization-challenge model. Our formulations conferred partial protection (significant delay in the time to reach 1% parasitemia) against challenge with the three chimeric parasites. Our results provide insights into the development of a vaccine targeting multiple strains of *P. vivax*.

## Introduction

Human malaria is caused by five different etiological agents, all belonging to the phylum Apicomplexa and the genus *Plasmodium: P. falciparum, P. vivax, P. ovale, P. malariae* and *P. knowlesi*. The latter is a non-human primate (NHP) parasite that causes infections in humans, including severe malaria^1^. The most prevalent species in the world are *P. falciparum* and *P. vivax*, both accounting for 95% of human malaria infections. Despite efforts to eliminate malaria from the world, the resistance of the main etiological agents to antimalarial drugs has increased considerably in recent years^2^, which has allowed malaria to spread in new areas and re-emerge in places where the disease has previously been considered eradicated^3,4^.

*P. falciparum* malaria is often considered the main target, as it causes the highest number of deaths among cases of infection. However, *P. vivax* malaria also causes severe symptoms and occasionally death^5^, and it is endemic in different regions of South and Central America, some parts of Africa and much of Asia. In 2019, 75% of malaria cases in the Americas were attributed to *P. vivax*, which has the highest geographical distribution and highest prevalence in the Americas among the etiological agents^6^. Research on *P. vivax* has long been neglected, resulting in limited knowledge of its biology, pathogenesis, and epidemiology compared to *P. falciparum*. Therefore, *P. vivax* is a relevant challenge to overcome for the success of malaria eradication programs^7^.

The main target of pre-erythrocytic malaria vaccines is the circumsporozoite protein (CSP), which covers the sporozoite surface. *P. vivax* CSP (*Pv*CSP) has two widely recognized variants, VK210 and VK247, which differ mainly in the sequence of repetitive amino acids in their central region. Each variant displays repeating nonapeptides, of which the more prevalent are GDRA(D/A)GQPA and ANGA(G/A) (C/D)QPG, respectively^8,9^. Nonetheless, several different peptide repeat motifs were described in the central region of both variants, and this genetic polymorphism could have impact on the efficacy of CSP-based vaccines^10^. A third variant from a parasite that causes *P. vivax* malaria in humans, called *Plasmodium vivax-*like, expresses CSP with APGANQ(E/G)GAA repeats (hereafter named *Pv*CSP-*P. vivax*-like) and was described in endemic regions of Papua New Guinea, Brazil, Indonesia and Madagascar^11^. Since the *P. vivax-*like parasite is among the *Plasmodium* species that infects NHP^12,13^ and the *Pv*CSP-*P. vivax*-like sequence is identical to *P. simiovale* CSP^14^, human infections with *P. vivax*-like parasites are commonly reported as cases of zoonoses^15,16^. However, when analyzing the genotype of parasites causing infections characterized microscopically as *P. vivax* in humans, a significant proportion of the parasites were the *P. vivax*-like variant, both in single infections and mixed with other *Pv*CSP allelic variants^17–19^. Moreover, the prevalence of this *P. vivax*-like variant is greater in regions of low incidence^18^, suggesting that if it is neglected, this variant might become an important reservoir of the disease. Other *Plasmodium* specie that commonly infects NHP and causes zoonotic *P. vivax* malaria in humans is *P. simium*, which shares high genetic identity with *P. vivax*^20^ and their two main CSP variants (VK210 and VK247) are identical^21^. Thus, a universal vaccine against all types of *P. vivax* malaria should include the VK210 and VK247 *P. vivax* variants and also the *P. vivax*-like variant.

Previously, we reported the generation of a recombinant protein fusing the repeat domains of the three *Pv*CSP variants (VK210, VK247 and *P. vivax*-like) in tandem, which contain immunodominant epitopes for B cells, and the conserved C-terminal region of *P. vivax* CSP (*Pv*CSP-All_CT_)^22^. Additionally, we generated two chimeric recombinant proteins containing the sequence of *Pv*CSP-All_CT_ fused to the mumps virus nucleocapsid protein to form nucleocapsid-like particles (NLPs) as a strategy to elicit strong and protective immune responses^23^. These recombinant proteins, NLP-CSP_CT_ and NLP-CSP_R_ (with and without the *Pv*CSP C-terminal region, respectively), were successfully produced in yeast *Pichia pastoris* and were highly immunogenic in mice when administered with Poly (I:C) adjuvant. Moreover, the immunization of mice with NLP-CSP_CT_/Poly (I:C) conferred partial protection against intradermal challenge^23^ with chimeric *P. berghei* parasites expressing the repetitive region of *Pv*CSP (VK210 allelic variant)^24^. Although these results were encouraging, the protective efficacy that these recombinant proteins potentially confer against the two other *Pv*CSP allelic variants, VK247 and *P. vivax*-like, remains to be elucidated.

To investigate whether the two recombinant *Pv*CSP proteins, NLP-CSP_R_ and NLP-CSP_CT_, can induce protective immune responses when combined with suitable adjuvants, we analyzed protective efficacy by immunizing mice followed by challenge with different chimeric *P. berghei* parasites expressing the three different *Pv*CSP variants. We have used two of these chimeric parasites in our established immunization-challenge model because they express full-length *P. vivax* CSP of VK210 and VK247 variants (*Pb-Pv*CSP210, *Pb-Pv*CSP247) on the sporozoite surface^25^. In this study, we generated a novel chimeric *P. berghei* parasite line that expresses the *Pv*CSP-*P. vivax*-like protein in sporozoites (*Pb-Pv*CSP-like G10). In addition, the protective efficacy of these recombinant proteins was analyzed in the presence of Poly (I:C) or Montanide ISA720 as adjuvants.

## Materials and methods

### Animals and ethics statements

Female inbred C57BL/6 (H-2b) mice were used to assess immunogenicity and protection after challenge. Tuck-ordinary (TO) outbred mice were used for parasite production and transmission. Mice were purchased from Harlan (UK). Female OF1 mice (6–7 weeks; Charles River, NL) were used to generate chimeric *P. berghei* lines. Immunogenicity and protection studies were performed in accordance with the recommendations of the UK Home Office Animals Act Project License. Procedures were approved by the University of Oxford Animal Care and Ethical Review Committee (PPL P9804B4F1).

Longevity assays were performed in accordance with the terms of the Guide for the Care and Use of Laboratory Animals of the Brazilian National Council of Animal Experimentation (http://www.cobea.org.br/). The protocol (CEUA/FCF no. 74.2016-P53) was approved by the Research Committee on Animal Experimentation of the School of Pharmaceutical Sciences of the University of São Paulo, Brazil.

Experiments for the generation of the chimeric *P. berghei* lines were granted with a license by the Competent Authority after advice on the ethical evaluation by the Animal Experiments Committee Leiden (AVD1160020171625). All experiments were performed in accordance with the Experiments on Animals Act, the applicable legislation in the Netherlands in accordance with the European guidelines (EU directive no. 2010/63/EU) regarding the protection of animals used for scientific purposes. The experiments were executed in a licensed establishment for the use of experimental animals (LUMC). Mice were housed in individually ventilated cages furnished with autoclaved aspen woodchip, fun tunnel, wood chew block and Nestlets at 21 ± 2 °C under a 12:12 h light–dark cycle with a relative humidity of 55 ± 10%.

This study was carried out in compliance with the ARRIVE (Animal Research: Reporting of In Vivo Experiments) guidelines for animals.

### Parasites

The following *P. berghei* ANKA reference parasite lines were used: (1) 1596cl1 (230p-GIMO_PbANKA_; RMgm-687, www.pberghei.eu), which contains a positive–negative selectable marker (SM) (human *dihydrofolate reductase:: yeast cytosine deaminase and uridyl phosphoribosyl transferase* (h*dhfr::yfcu*)) cassette integrated into the silent *230p* gene locus (PBANKA_030600)^26^; (2) the wild-type (WT) reference line cl15cy1 of *P. berghei* ANKA^27^ and the reporter *Pb*ANKA parasite line *Pb*GFP-Luc_con_ (676m1cl1). The *Pb*GFP-Luc_con_ parasite expresses a GFP (mutant3) and firefly luciferase (LUC-IAV) fusion protein from the constitutive *eef1a* promoter and is selectable marker (SM)-free^27^. The reporter cassette is integrated into the neutral *230p* locus (PBANKA_030600). For details of *Pb*GFP-Luc_con_, see RMgmDB entry #29 (http://www.pberghei.eu/index.php?rmgm=29).

In addition, we used two existing chimeric *P. berghei* lines in which the *P. berghei csp* gene was replaced with either the *P. vivax csp* VK210 allele or the *P. vivax* VK247 allele (*Pb-Pv*CSP210, https://www.pberghei.eu/index.php?rmgm=4136; *Pb-Pv*CSP247, https://www.pberghei.eu/index.php?rmgm=4137)^25^.

### Generation and genotyping of chimeric *P. berghei* lines expressing the PvCSP-*P. vivax*-like protein

To generate the chimeric *P. berghei* replacement line, we replaced the *P. berghei csp* coding sequence (CDS; *Pbcsp*; PBANKA_0403200) with the *Pv*CSP-*P. vivax*-like CDS (Locus PVU09738, Accession U09738) using a 2-step GIMO transfection protocol^25,26,28^. In the first step, we deleted the *P. berghei csp* CDS and replaced it with a positive–negative selectable marker to create a *P. berghei csp* deletion GIMO line (*Pb*ANKA-CSP GIMO). The construct (pL1929) used and the generation of the *Pb*ANKA-CSP GIMO line (line 2251cl1) have been described previously^29^. This construct contains the positive–negative (h*dhfr*::y*fcu)* SM cassette and was used to insert both the *Pbcsp* 5′ and 3′ gene targeting regions (TRs), encompassing the full-length promoter and transcription terminator sequences, respectively, and was transfected into *Pb*GFP-Luc_con_ parasites (676m1cl1) using standard transfection methods^30^. Transfected parasites were selected in mice by applying positive selection by providing pyrimethamine in the drinking water^30^. Transfected parasites were cloned using the limiting dilution method^31^, resulting in the *Pb*ANKA-CSP GIMO line (line 2251 cl1). In the second step, we replaced the positive–negative SM in the *Pb*ANKA-CSP GIMO genome with the *Pv*CSP-*P. vivax*-like CDS by GIMO transfection to create the *P. berghei* chimeric *Pb-Pv*G10 replacement line *Pb-Pv*G10(r). This line was obtained by modifying the construct used in the first step (pL1929); specifically, the h*dfhr*::y*fcu* SM cassette was removed and replaced with the *Pv*CSP-*P. vivax*-like CDS, generating plasmid pL2161. The *Pv*CSP-*P. vivax*-like CDS was ordered from GeneArt Gene Synthesis—Thermo Fisher Scientific. The pL2161 construct was sequenced to ensure that no mutations were present in the *Pv*CSP-*P. vivax*-like CDS during the cloning process. The construct was linearized using AflII and SacI restriction enzymes outside of the 5’ and 3’ TRs before transfection. The construct was used to transfect parasites of the PbANKA-CSP GIMO line (line 2251 cl1)^26^ using standard methods of GIMO transfection to generate a single replacement gene chimeric parasite^25,32–34^. Transfected parasites were selected in mice by applying negative selection by providing 5-fluorocytosine (5-FC) in the drinking water^35^. Negative selection results in the selection of chimeric parasites where the h*dhfr*::y*fcu* SM in the *csp* locus on chromosome 4 of the PbANKA-CSP GIMO line is replaced with the *Pv*CSP-*P. vivax*-like CDS*.* Selected chimeric parasites were cloned using the limiting dilution method^31^. Correct integration of the constructs into the genome of chimeric parasites was analyzed by performing a gDNA Southern analysis of pulsed field gel (PFG)-separated chromosomes, as previously described^36^. This method creates chimeric ‘gene replacement’ *P. berghei* parasites that lack the *Pbcsp* CDS but express the *Pv*CSP-*P. vivax*-like protein (*Pb-Pv*G10(r); line 2710 cl1) under the control of the *P. berghei csp* regulatory sequences.

The *Pv*CSP-*P. vivax*-like Gabon Clone G10 CDS (Locus PVU09738, Accession U09738) gene was introduced into the genome as an additional copy of the gene in the neutral *230p* locus using the previously described ‘gene insertion/marker out’ (GIMO) technology^26,28,37^ and the standard GIMO DNA construct pL0043 to generate the chimeric *P. berghei* additional copy line. This construct contains 5’ and 3’ targeting sequences for the *230p* locus, as well as a multiple-cloning site for the integration of transgene expression cassettes. This construct integrates transgenes by double crossover homologous recombination and replaces the positive–negative SM (human *dihydrofolate* reductase:: yeast *cytosine deaminase* and *uridyl phosphoribosyl transferase* (hdhfr::y*fcu*)) cassette with the transgene expression cassette. The expression cassette contained the *Pv*CSP-*P. vivax*-like CDS flanked by the 5’ and 3’ promoter and transcription terminator sequences of the *P. berghei uis4* gene (PBANKA_0501200), which were amplified from *P. berghei* ANKA WT genomic DNA^36^. The coding sequence of the *P. vivax CSP-like G10* gene (Locus PVU09738, Accession U09738) was ordered from GeneArt Gene Synthesis—Thermo Fisher Scientific. In addition, a reporter cassette containing GFP::luciferase^30^ driven by the constitutive *P. berghei elongation factor 1 alpha* (ef1α) promoter was also cloned into the transgene construct to generate the gene insertion construct pL2163 (*PvCSP-Like G10@*_*Pbuis4*_ + *GFP::Luc@Pbeef1a_230p*) targeting the neutral *230p* locus on chromosome 3. The coding sequence and promoter region of the construct were confirmed by sequencing.

The pL2163 construct was linearized by SacII restriction digestion and introduced into parasites of the GIMO motherline 1596cl1 using standard methods of GIMO transfection^26^. Transfected parasites were selected in mice through the addition of 5-fluorocytosine (5-FC) to the drinking water^35^, resulting in negative selection of parasites in which the SM in the *230p* locus was replaced by the *Pv*CSP-*P. vivax*-like expression/reporter cassette. The selected chimeric parasites were cloned using the limiting dilution method^31^. Primer sequences are listed in Table S1. Correct integration of the *Pv*CSP-*P. vivax*-like coding sequence (under control of the *Pbuis4* promoter) into the genome of clones of the chimeric line (*Pb-Pv*CSP-like G10, 2700 cl1) was analyzed by performing a diagnostic PCR analysis of gDNA and Southern analysis of pulsed field gel (PFG)-separated chromosomes^36^.

### Phenotype and fitness assessment of the chimeric *P. berghei* lines expressing PvCSP-*P. vivax*-like protein

Multiplication of blood stages in mice was determined during the cloning period as previously described^,38^. Feeding of *Anopheles stephensi* mosquitoes, determination of oocyst production, and sporozoite (spz) collection were performed as described elsewhere^36,38^. The infectivity of chimeric spz was assessed by determining the T1% period (i.e., the time to reach 1% parasitemia) after an intravenous injection of 1000 spz in the tail vein of inbred BALB/c mice (Harlan, UK).

The expression of the *Pv*CSP-*P. vivax*-like protein in spz was analyzed by performing an immunofluorescence assay (IFA) using sera from mice immunized with the recombinant proteins (diluted 1:100). As a control, the 3D11 antibody^39^ recognizing *P. berghei* CSP was used (diluted 1:1000). Purified spz were fixed with 4% paraformaldehyde in PBS for 20 min on ice, washed three times with PBS and blocked with 20 µl of 10% FCS + 1% BSA in PBS for 30 min at room temperature. Excess blocking medium was removed, followed by the addition of 20–25 µL of primary monoclonal antibody in PBS containing 10% FCS + 1% BSA (blocking medium) for 1–2 h at room temperature or overnight at 4 °C. After the incubation, the primary antibody was removed, and the slides were washed three times with PBS, followed by staining with the secondary antibody (Alexa Fluor-488 goat anti-mouse IgG from Life Technologies, Cat# A-11001) diluted 1:800 in PBS containing 10% FCS + 1% BSA (blocking medium) for 1 h at room temperature. After three washes with PBS, nuclei were stained with 2% Hoechst-33342 (Cell Signaling Technology #4082S) in PBS for 10 min at room temperature, washed twice with PBS and air-dried, followed by the addition of fluorescence mounting medium (Dako, code S3023). Cover slips were mounted onto the slides, and the slides were sealed with nail polish and allowed to dry overnight in the dark as described in a previous study^37^. The spz were analyzed using a DMI-300B Leica fluorescence microscope in both blue and green channels, and images were processed using ImageJ software.

### Purified recombinant proteins obtained after expression in *P. pastoris*

Expression and purification of recombinant CSP proteins from *P. pastoris* was carried out as described previously^23^. Briefly, yeast clones containing the previously selected plasmids of interest were cultured for 24 h at 30 °C under constant stirring (230 rpm) in 40–200 mL of 3% glycerol-containing medium (BMGY). Cells were then harvested by centrifugation, solubilized in 40–200 mL of medium containing 1.0% methanol (BMMY) and cultured at 28 °C with constant stirring (230 rpm). Induction was maintained by the daily addition of 1.0% methanol. After 72–96 h of incubation, the cells were removed by centrifugation, and the supernatants were filtered through 0.45 µm membranes (Millipore). Recombinant proteins were then purified by affinity and ion exchange chromatography using a HisTrap FF column and Q-Sepharose resin, respectively, both coupled to the ÄKTAprime system (GE Healthcare). Fractions containing the highly pure recombinant proteins were collected and dialyzed against PBS.

### Mouse immunization protocol

Groups of six female C57BL/6 mice aged 6–8 weeks were subcutaneously (s.c.) immunized thrice with the corresponding formulation of recombinant protein/adjuvant. For each dose, a final volume of 100 µl (10 µg of protein/sterile PBS/adjuvant) was injected at the base of the tail of each mouse. The adjuvants used were Poly (I:C) HMW (InvivoGen, 50 μg per dose per mouse in ratio 50/50 protein/adjuvant) and Montanide ISA720 (Seppic, emulsion in ratio 70/30 protein/adjuvant). The formulations were prepared just before administration.

### Antibody measurements

Twelve days after each immunization, blood was collected from the tail vein, and sera were analyzed for the presence of antibodies recognizing each recombinant protein. Antibodies were detected by enzyme-linked immunosorbent assay (ELISA), essentially as described in a previous study^22^. The recombinant proteins NLP-CSP_CT_, NLP-CSP_R_^23^, y*Pv*CSP-VK210_CT_, y*Pv*CSP-VK247_CT,_ and y*Pv*CSP-*P. vivax*-like _CT_^22^ were employed as solid phase-bound antigens (200 ng/well). After an overnight incubation at RT, plates were washed with a solution of PBS containing 0.05% Tween-20 (PBS-T) and blocked with a blocking solution (PBS, 5% (w/v) skimmed milk) for 2 h at 37 °C. Serial dilutions of murine polyclonal sera were added to the wells and incubated for 1 h at RT; after washes with PBS-T, peroxidase-labeled goat anti-mouse IgG (Sigma, St. Louis, USA), diluted 1:3000, was added to each well. Reactions were developed with the OPD/acid stop system. Anti-IgG titers were determined based on the highest dilution of sera yielding an A_492_ greater than 0.1.

### Challenge of mice with chimeric sporozoites

Spz of the chimeric parasite lines *Pb-Pv*CSP-210 (2196cl1), *Pb-Pv*CSP247 (2199cl1)^25^ and *Pb-Pv*CSP-like G10 (2700cl1) were used to challenge immunized mice. Female *A. stephensi* mosquitoes were used to produce chimeric spz. After 21 days of incubation in a humidified incubator at 19–21 °C on a 12-h day-night cycle and feeding on a fructose-*p*-aminobenzoic acid (PABA) solution, the mosquitoes were dissected, salivary glands were isolated, and spz were extracted. The total number of spz was determined using a hemocytometer, and 2000 spz were intravenously (i.v.) injected in 100 µL 14 days after the second booster immunization.

### Parasitemia analyses

Thin blood smears were prepared daily from day 4 to day 12 after challenge or until the day after mice reached 1% parasitemia. The smears were prepared on glass slides with a drop of blood obtained from mouse tail veins, fixed with methanol and stained for 15 min using 10% Giemsa. The glass slides were observed under a light microscope, and the percentages of parasitized red blood cells were determined. The time required to reach 1% parasitemia (T1%) is a variable calculated by a linear regression equation using the percentage of parasites detected in blood on the first three consecutive days with positive parasitemia. Protection analysis using T1% period as index is a useful tool to assess vaccine efficacy, as the comparative time to reach a determined level of parasitemia reflects the prepatent period and the number of parasites erupting from the liver^40^.

### Statistical analyses

All analyses and graphics were performed/generated using GraphPad Prism version 8.0 (GraphPad Software Inc., La Jolla, CA, USA). IgG Ab titers were compared using one-way analysis of variance (ANOVA). One-way ANOVA was also used to compare normally distributed log-transformed means for the different animal groups. Multiple comparisons were assessed using Tukey’s posttest with a significance level of p < 0.05. Survival curves were compared using a log-rank Mantel-Cox test with a significance level of p < 0.05.

## Results

### ***Pv***CSP-specific antibody responses in mice immunized with the recombinant proteins NLP-CSP_CT_ and NLP-CSP_R_

**Figure 1:**
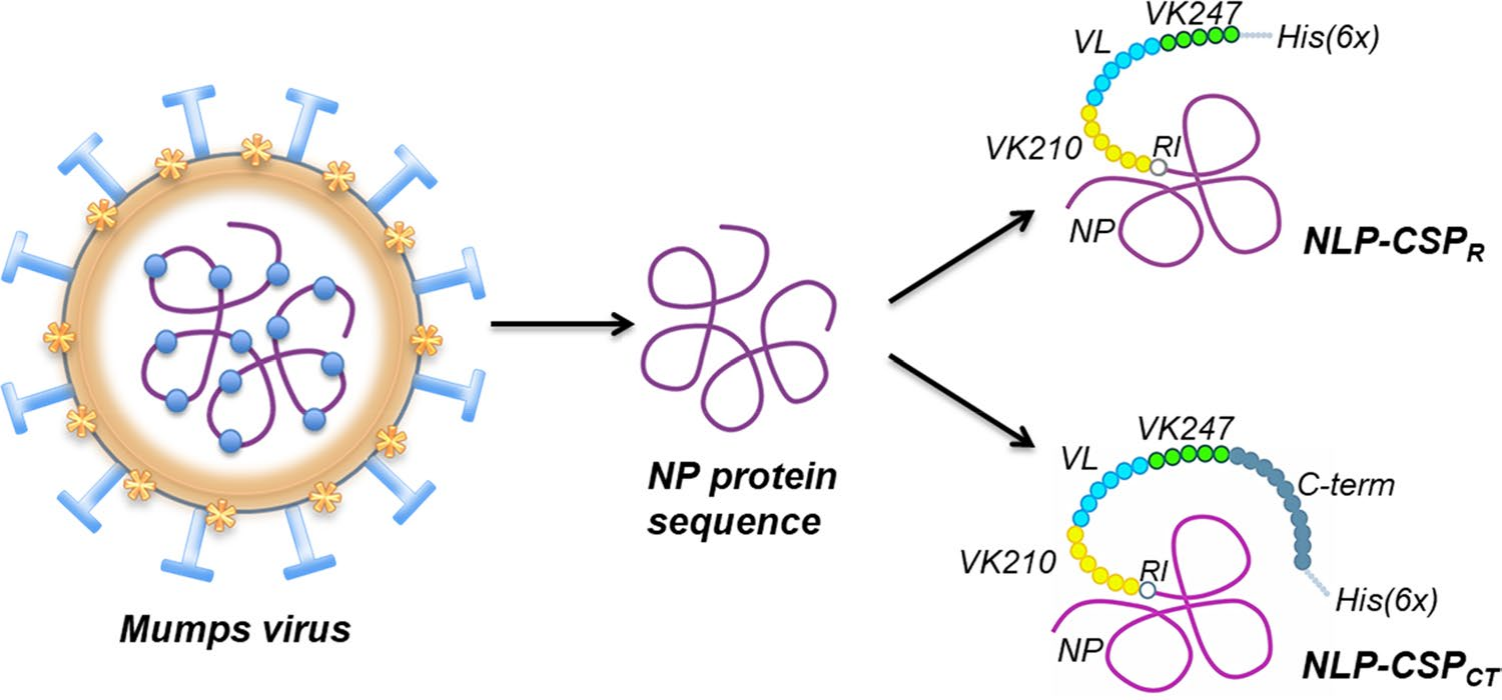
Schematic representation of mumps virus and NLP-CSP proteins. Mumps virus proteins are represented in the left panel. From outside to inside: Hemagglutinin-Neuraminidase protein (light blue) and Fusion protein (gold) in the membrane surface; Matrix protein (orange) and Nucleocapsid protein (purple) with associated RNAs (blue) in the inside. NLP-CSPs proteins are represented in the right panels. NP sequence is represented in purple, *Pv*CSP-RI sequence in withe circle, *Pv*CSP-VK210 repeats in yellow circles, *Pv*CSP-*P. vivax*-like (VL) in cyan circles, *Pv*CSP-VK247 in green circles, *Pv*CSP-C-terminal sequence in dark grey and the His-tag sequence in light grey.

In previous studies, we generated two chimeric recombinant proteins, NLP-CSP_CT_ and NLP-CSP_R_, fusing domains of the three *Pv*CSP variants (VK210, VK247, and *P. vivax*-like) to the mumps virus nucleocapsid protein (NP)^23^. A schematic representation of the mumps virus, the NP protein and the new recombinants is depicted in Fig. 1. Briefly, the strategy used was fusing the malaria antigens to a core-viral protein rather than the surface proteins, thus avoiding the possible interference of immunological memory against mumps virus in the general population^23^.

**Figure 2:**
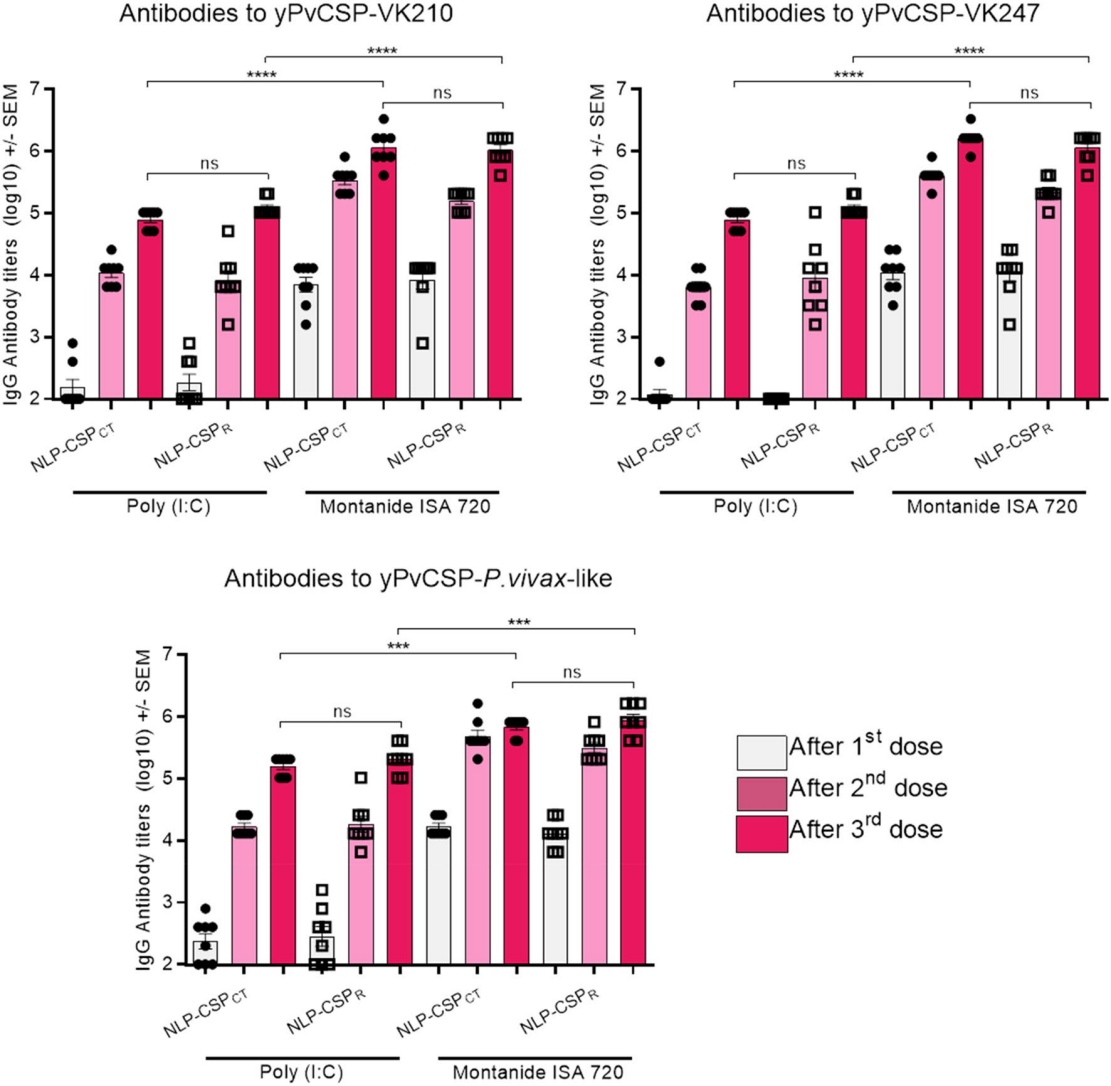
Humoral immune response in mice immunized with recombinant proteins. C57BL/6 mice were s.c. immunized with the recombinant NLP-CSP_CT_ and NLP-CSP_R_ proteins in the presence of Poly (I:C) or Montanide ISA 720 adjuvants using the scheme shown in Table 1. IgG antibody titers were determined using ELISA assay at 12 days after the administration of each dose using the individual PvCS proteins (y*Pv*CSP-VK210, y*Pv*CSP-VK247 and y*Pv*CSP-*P. vivax*-like) as solid-phase bound antigens.

First, we compared the humoral immune response induced by immunizing mice with different vaccine formulations. Groups of six C57BL/6 female mice were immunized with 10 μg of the two recombinant proteins, NLP-CSP_CT_ or NLP-CSP_R_, mixed with either Poly (I:C) (50 μg/dose) adjuvant or emulsified in Montanide ISA720 (7:3), an oil–water emulsion. Each animal received three immunizations 14 days apart in a homologous prime-boost vaccination regimen (Table 1). The antibody titers against each recombinant *Pv*CSP variant (y*Pv*CSP-VK210, y*Pv*CSP-VK247, y*Pv*CSP-*P. vivax*-like) were measured by ELISA twelve days after the administration of each dose. As shown in Fig. 2, no statistically significant difference was observed in the antibody titers elicited by the two recombinant proteins (NLP-CSP_CT_ or NLP-CSP_R_) when combined with the same adjuvant (NLP-CSP_CT_ or NLP-CSP_R_ in the presence of Poly (I:C) for y*Pv*CSP-VK210 p = 0.9795, y*Pv*CSP-VK247 p = 0.9608 and y*Pv*CSP-*P. vivax*-like p = 0.9994, and NLP-CSP_CT_ or NLP-CSP_R_ in the presence of Montanide ISA 720 for y*Pv*CSP-VK210 p > 0.9999, y*Pv*CSP-VK247 p = 0.9925 and y*Pv*CSP-*P. vivax*-like p = 0.9994).

**Table 1:** Groups of immunized C57BL/6 female mice.

Group (n = 6 mice/group)	Prime (day 0)	Boost 2x (days 14 and 28)	Adjuvant
G1	–	–	Poly (I:C)
G2	NLP-CSP_CT_	NLP-CSP_CT_	Poly (I:C)
G3	NLP-CSP_R_	NLP-CSP_R_	Poly (I:C)
G4	–	–	Montanide ISA720
G5	NLP-CSP_CT_	NLP-CSP_CT_	Montanide ISA720
G6	NLP-CSP_R_	NLP-CSP_R_	Montanide ISA720

In contrast, significant differences in IgG titers were observed when we compared the effects of the different adjuvants. With the Poly (I:C) adjuvant, the titers of IgG specific for all three variants were detectable (~ 10^4^) in both immunized groups only after the administration of two doses. With the administration of the third dose, IgG titers were greater than 10^5^ in all immunized mice. When the same analysis was performed for groups immunized with Montanide ISA 720, antibody titers reaching 10^4^ levels were detected after the administration of a single dose. Moreover, after the administration of the second and third doses, even higher IgG titers (10^6^) against all three *Pv*CSP variants were detected (Fig. 2).

When antibody titers were compared after the administration of three doses using the same recombinant protein but with a different adjuvant, all groups of mice immunized with Montanide ISA 720 had significantly higher specific IgG titers than mice immunized with Poly (I:C) (NLP-CSP_CT_ to y*Pv*CSP-VK210 p > 0.0001, y*Pv*CSP-VK247 p > 0.0001 and y*Pv*CSP-*P. vivax*-like p = 0.0004, and NLP-CSP_R_ to y*Pv*CSP-VK210 p > 0.0001, y*Pv*CSP-VK247 p > 0.0001 and y*Pv*CSP-*P. vivax*-like p = 0.0004).

We also analyzed the longevity of the IgG antibodies. As shown in Suppl. Figure 1, *Pv*CSP-specific IgG titers remained higher than 10^4^ in the Poly (I:C) adjuvant-treated groups and on the order of 10^5^ in the Montanide adjuvant-treated groups for at least 102 days after priming. These responses were antigen-specific, since mice immunized only with either adjuvant did not elicit detectable *Pv*CSP-specific IgG antibodies at any time point analyzed (Suppl. Figure 1).

It is worth mentioning that any potential interference of yeast components in the specificity of the produced antibodies was discarded in previous studies, by using recombinant proteins produced in bacteria as solid phase-bound antigens^22,23^.

### Preclinical immunization-challenge model: generation of a chimeric *P. berghei* line expressing *P. vivax*-like CSP in sporozoites (*Pb-Pv*CSP-like G10)

In previous studies, we generated two chimeric *P. berghei* parasites (*Pb-Pv*CSP210 and *Pb-Pv*CSP247) that express the full-length VK210 and VK247 variants of *P. vivax* CSP on the spz surface^25^. These parasites have been used to analyze protective efficacy in mice immunized with different vaccine candidates targeting *Pv*CSP with an immunization-challenge mouse model^41–43^. To analyze protective immune responses induced by the two recombinant proteins NLP-CSP_CT_ or NLP-CSP_R_ against not only the VK210 and VK247 repeats but also against the *P. vivax*-like variant, we generated a third chimeric *P. berghei* parasite that expressed the *Pv*CSP-*P. vivax*-like protein in spz.

**Figure 3:**
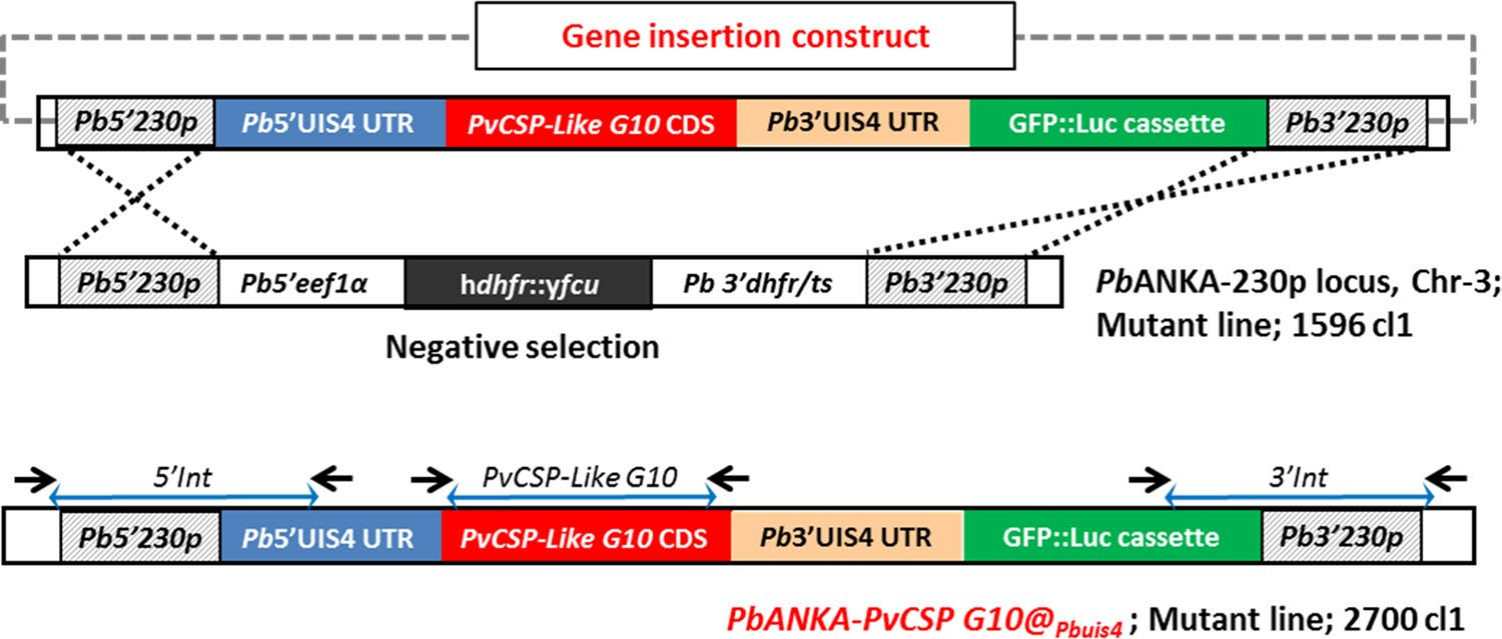
Strategy to generate a chimeric *P. berghei* parasite line expressing a *PvCSP-P. vivax-like* protein as additional CSP. An additional copy line in which the *Pv*CSP-*P. vivax*-like CDS (PVU09738) gene was introduced into the genome as an additional copy of the gene in the neutral *230p* locus. The construct that contains the ‘*Pv*CSP-*P. vivax*-like gene expression cassette’ was integrated into the *230p* locus on chromosome 3 of the *P. berghei* ANKA GIMO mother line by GIMO transfection using negative selection (5-FC), resulting in the expression of the *Pv*CSP-*P. vivax*-like gene under the control of the *Pbuis4* gene promoter and transcriptional terminator sequences. This construct also expresses a GFP and firefly luciferase (LUC-IAV) fusion protein under control of the constitutive *Pbeef1a* promoter and is selectable marker (SM)-free. The construct is integrated into the neutral *p230p* locus by double crossover integration. Black arrows: location of PCR primers used for the diagnostic PCR analysis.

**Figure 4:**
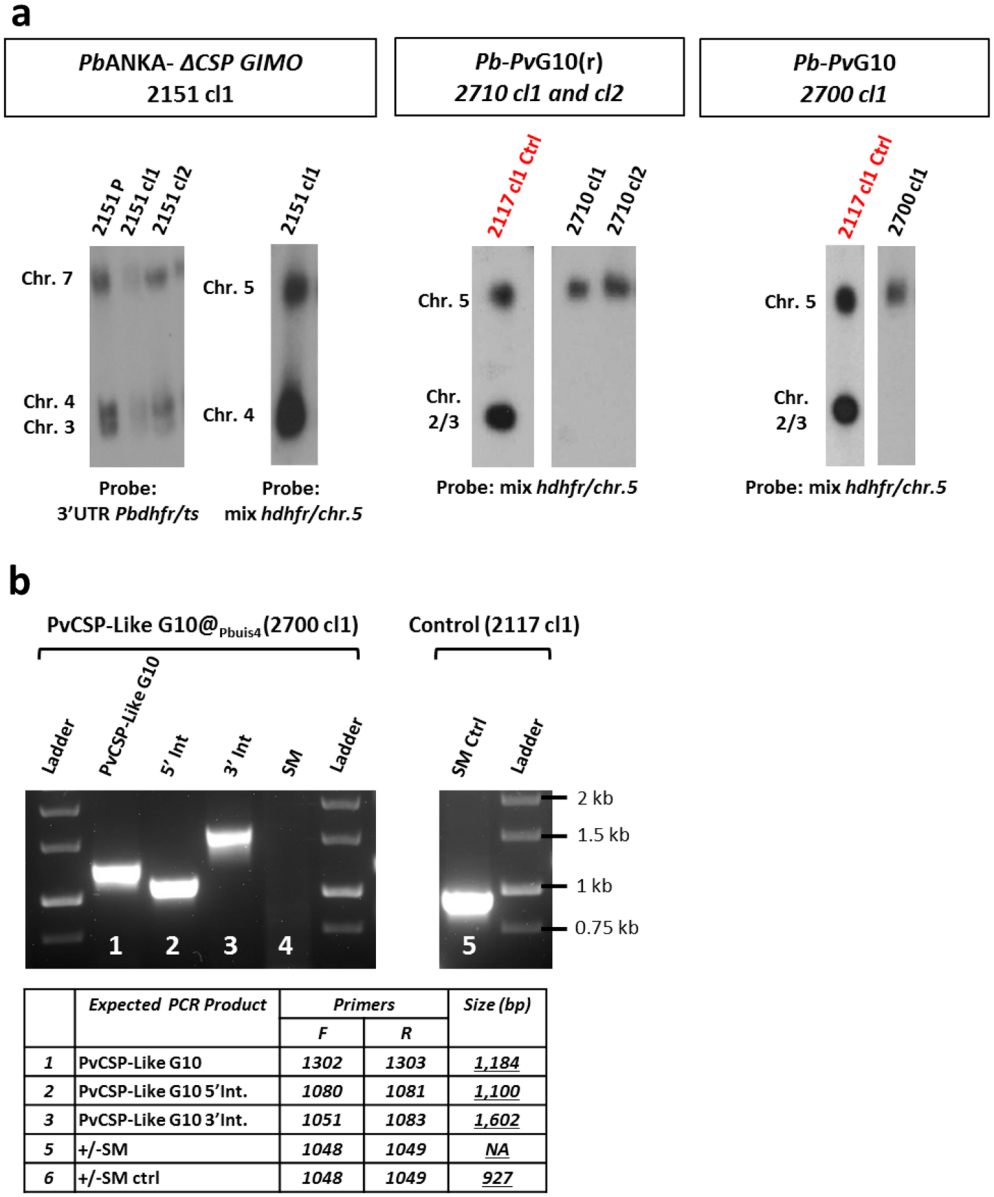
Genotyping analyses of chimeric *P. berghei* parasite lines expressing the *Pv*CSP-*P. vivax*-like protein. Cropped gels and blots are displayed in this figure. Full-length blots/gels are presented in Supplementary Fig. 4. (**a**) Genotyping analysis of the replacement line *Pb-Pv*G10(r) (2710cl1 and cl2) and its intermediate GIMO mother-line (2151cl1) using a Southern analysis of chromosomes (chr.) separated by pulsed-field gel electrophoresis (PFGE) and diagnostic PCR analysis. Left panel: Hybridization of chr. from line 2151cl1 with the 3′UTR *Pbdhfr/ts* confirms integration of construct pL1929 into the *Pbcsp* gene on chr. 4. In addition, this probe hybridizes to the GFP-Luc reporter cassette on chr. 3 and to the endogenous *Pbdhfr/ts* on chr. 7. The correct integration of the SM is also confirmed by using a mixture of two probes: one recognizing hdhfr and a control probe recognizing chr. 5. Middle panel: The correct integration of the *Pv*CSP-*P. vivax*-like gene expression construct (pL2161) into the GIMO locus was confirmed by showing the removal of the h*dhfr::*y*fcu* selectable marker (SM) cassette in clones of the chimeric parasite line *Pb-Pv*G10(r) (2710cl1 and 2710cl2). The Southern blot was hybridized with a mixture of two probes: one recognizing h*dhfr* and a control probe recognizing chr. 5. As an additional control (ctrl), parasite line 2117cl1 was used with the h*dhfr::*y*fcu* SM integrated into chr. 3. Right panel: Southern analysis of chr. of the ‘additional copy’ chimeric line *Pb-Pv*G10(r) (2700cl1) confirms the correct integration of the expression PvCSP-Like G10@Pbuis4 construct (pL2163) into the GIMO locus (*230p* on chr. 3), shown as the removal of the h*dhfr::*y*fcu* SM cassette in cloned chimeric parasites compared to a control probe recognizing chr. 5. As an additional control (Ctrl), parasite line 2117cl1 is also shown, as it retains h*dhfr::*y*fcu* SM in the *230p* locus on chr. 3. (**b**) Genotyping using a diagnostic PCR analysis of the chimeric *Pb-Pv*CSP-like G10 line (2700cl1; left panel) confirms correct integration of the *PvCSP-Like@Pbuis4* expression cassette. Correct integration is shown by the absence of the h*dhfr*::y*fcu* SM and the presence of the *Pv*CSP-*P. vivax*-like CDS and the correct integration of the construct into the genome at both the 5′ and 3′ regions (5′int and 3′int; see Fig. 3 for primer locations). The primer sequences used in this study are shown in Table S1, while the expected PCR product sizes and the primer numbers are listed in the table below the PCR analysis. As an additional control (ctrl), parasite line 2117cl1 (right panel) was used to validate the primers used to amplify the h*dhfr::*y*fcu* SM that was integrated into chr. 3 but has been removed from 2700cl1.

We first generated a chimeric *P. berghei* ANKA line in which the endogenous *P. berghei csp* gene was replaced with the *Pv*CSP-*P. vivax*-like gene using the same GIMO transfection approach that was used to generate the chimeric *Pb-Pv*CSP210 and *Pb-Pv*CSP247 lines (Suppl. Figure 2 and Fig. 4a)^25^. Two independent clones of this *Pb-Pv*G10(r) line (2710cl1, 2710cl2) produced normal numbers of oocysts. However, in contrast to the other two chimeric lines that express *Pv*CSP-VK210 and *Pv*CSP-VK247, the parasites expressing the *Pv*CSP-*P. vivax*-like protein did not form visible spz inside oocysts, and only very few salivary gland spz were observed (Table S2). The cause of the lack of sporozoite formation is unclear, but the CSP of different *Plasmodium* species do not always fully complement the function of the CSP of other species. For example, chimeric *P. falciparum* replacement lines expressing *P. vivax* CSP (VK210 and VK247) also have a defect in the formation of spz^44^. An alternative strategy is the generation of rodent-infectious *P. berghei* and *P. falciparum* spz which can be engineered to express CSP proteins on the spz surface from two different *Plasmodium* species^45–47^. Based on this observation, we decided to generate chimeric *P. berghei* spz expressing both the *Pb*CSP and the *Pv*CSP-*P. vivax*-like protein. In this chimeric line (*Pb-Pv*CSP-like G10, 2700 cl1), the *Pv*CSP-*P. vivax*-like gene is introduced into the genome as an additional copy of the csp gene in the neutral *230p* locus using GIMO transfection (Figs. 3, 4a,b). The *Pv*CSP-*P. vivax*-like gene is flanked by the 5′ and 3′ promoter and transcription terminator sequences of the *P. berghei uis4* gene, which is specifically expressed in spz and liver stages^48^. *Pb-Pv*CSP-like G10 parasites showed normal asexual blood stage multiplication in mice (data not shown), and both oocyst and sporozoite production in *A. stephensi* mosquitoes was comparable to wild-type *P. berghei* parasites (Table S2). The infectivity of chimeric spz, as determined by the length of the T1% period after an intravenous injection of 1000 spz in BALB/c mice, was similar to the T1% period in mice infected with wild-type *P. berghei* spz (Table S2). These results demonstrate that the chimeric *Pb-Pv*CSP-like G10 parasites produce fully infectious spz that are able to complete liver stage development in mice. The expression of the *Pv*CSP-*P. vivax*-like protein in *Pb-Pv*CSP-like G10 spz was determined by immunofluorescence analysis using sera from mice immunized with the recombinant proteins. The 3D11 antibody recognizing *P. berghei* CSP was used as a control. *Pb-Pv*CSP-like G10 spz stained both with the antiserum and the 3D11 antibody (Suppl. Figure 3), demonstrating the expression of both *P. berghei* CSP and *Pv*CSP-*P. vivax*-like protein in spz of the *Pb-Pv*CSP-like G10 line.

### Protective efficacy in mice immunized with different vaccine candidates targeting *Pv*CSP variants using the immunization-challenge mouse model

**Figure 5:**
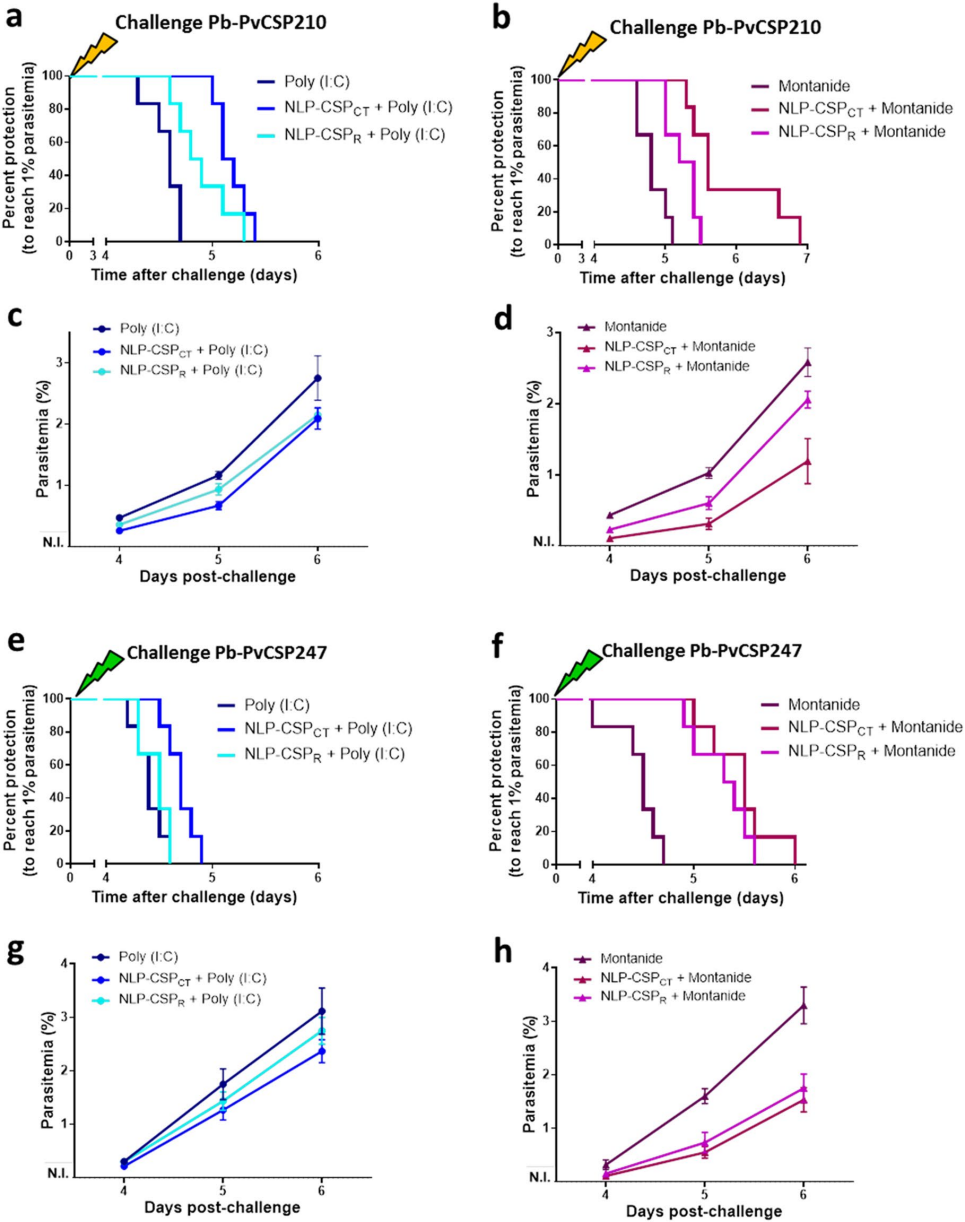

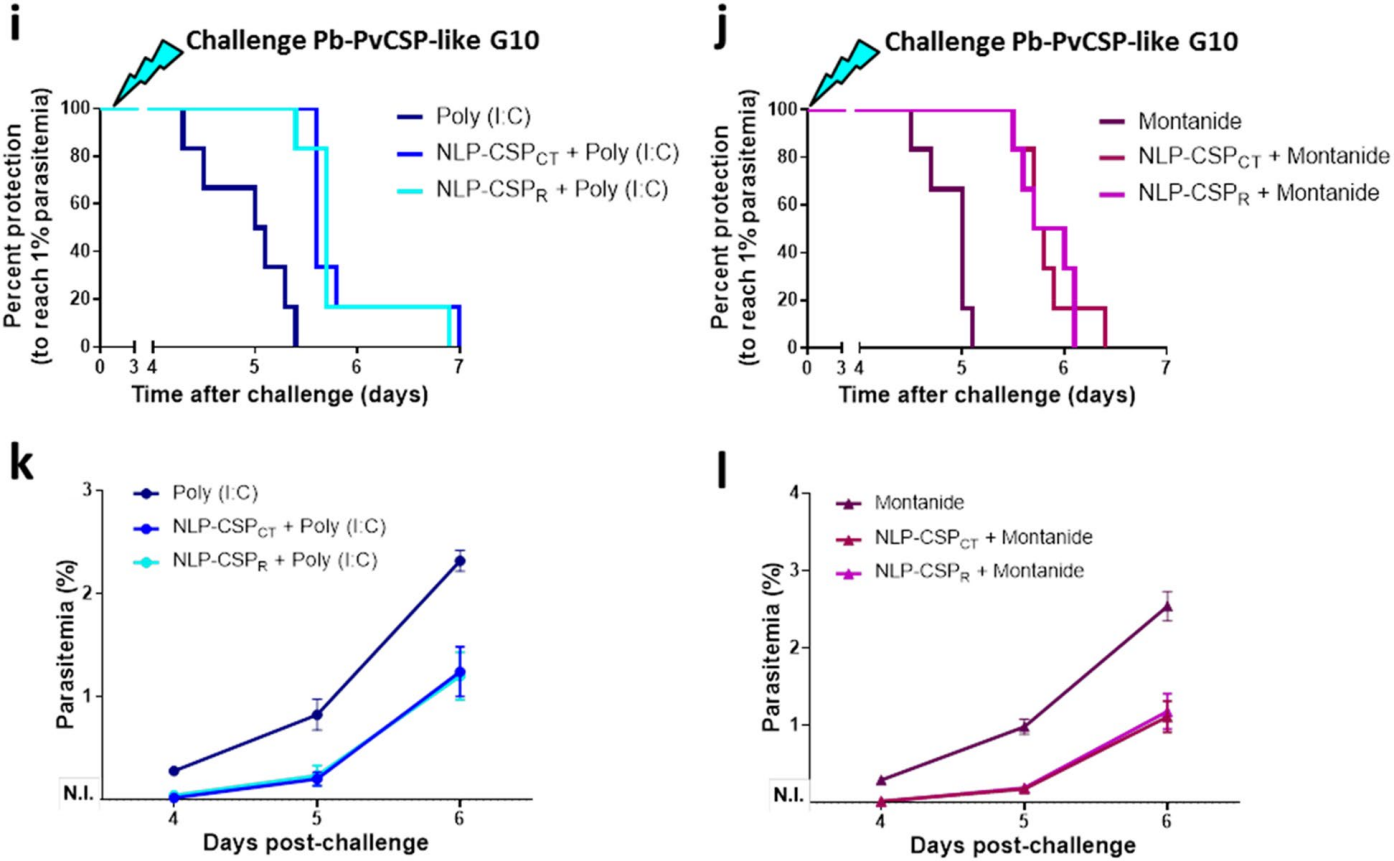
T1% period in immunized mice after challenge with *Pb*-*Pv*CSP transgenic sporozoites. Groups of six C57BL/6 mice were s.c. immunized with the vaccine formulations in the presence of Poly (I:C) or Montanide adjuvants, following the scheme shown in Table 1. Upper panel: Immunized mice were challenged 14 days after the third dose with 2,000 *Pb*-*Pv*CSPVK210 sporozoites. Percent protection to reach 1% parasitemia (**a**, **b**) and follow-up analysis of parasitemia at days 4, 5 and 6 after challenge (**c**, **d**) is shown. Middle panel: Immunized mice were challenged 14 days after the third dose with 2,000 *Pb*-*Pv*CSPVK247 sporozoites. Percent protection to reach 1% parasitemia (**e**, **f**) and follow-up analysis of parasitemia at days 4, 5 and 6 after challenge (**g**, **h)** is shown. Lower panel: Immunized mice were challenged 14 days after the third dose with 2000 spz of the new chimeric *Pb-Pv*CSP-like G10 parasite. Percent protection to reach 1% parasitemia (**i**, **j**) and follow-up analysis of parasitemia at days 4, 5 and 6 after challenge (**k**, **l**) is shown. Significant differences in T1% periods (see Table 2) were analyzed by applying the log-rank (Mantel-Cox) test.

To analyze protective immune responses induced in the mice by immunization with the recombinant proteins NLP-CSP_R_ and NLP-CSP_CT_, we challenged mice with spz of the three chimeric *P. berghei* lines, *Pb-Pv*CSP210, *Pb-Pv*CSP247 and *Pb-Pv*CSP-like G10. The results are shown in Fig. 5; *Pb-Pv*CSP210 challenge in the upper panel (Fig. 5a–d), *Pb-Pv*CSP247 challenge in the middle panel (Fig. 5e–h) and *Pb-Pv*CSP-like G10 challenge in the lower panel (Fig. 5i–l).

Groups of six C57BL/6 mice were immunized as described in Table 1 and challenged 2 weeks after the last immunization by administering an intravenous (i.v.) injection of 2000 spz *Pb-Pv*CSP210. The protective efficacy was assessed by determining the T1% period after challenge (Fig. 5a, b) by following parasitemia (Fig. 5c, d); p values denoting significant differences in the T1% period between control (adjuvant) and immunized mice are depicted in Table 2. With either adjuvant, a statistically significant delay in the T1% was observed in mice immunized with NLP-CSP_CT_ and NLP-CSP_R_ compared to the adjuvant-only groups. However, a significant difference between the protective efficacy of mice immunized with NLP-CSP_CT_ or NLP-CSP_R_ was only observed in the Montanide adjuvant-treated groups (p = 0.0192, Table 2), showing higher protective efficacy of the NLP-CSP_CT_ protein.

**Table 2:** Prepatent (T1%) periods and statistical significance of protective efficacy.

Challenge	Comparison	Poly (I:C)			Montanide ISA720		
		NLP-CSP_CT_ vs. Adj	NLP-CSP_R_ vs. Adj	NLP-CSP_CT_ vs. NLP-CSP_R_	NLP-CSP_CT_ vs. Adj	NLP-CSP_R_ vs. Adj	NLP-CSP_CT_ vs. NLP-CSP_R_
*Pb-PvCSP210*	Median T1%	5.15 vs. 4.6	4.85 vs. 4.6	5.15 vs. 4.85	5.6 vs. 4.8	5.3 vs. 4.8	5.6 vs. 5.3
	p values	p = 0.0008	p = 0.0136	n.s. p = 0.1026	p = 0.0005	p = 0.0068	p = 0.0192
*Pb-PvCSP247*	Median T1%	4.7 vs. 4.4	4.5 vs. 4.4	4.7 vs. 4.5	5.5 vs. 4.5	5.35 vs. 4.5	5.5 vs. 5.35
	p values	p = 0.0049	n.s. p = 3797	p = 0.0136	p = 0.0005	p = 0.0005	n.s. p = 0.2798
*Pb-PvCSP-like G10*	Median T1%	5.6 vs. 5.05	5.7 vs. 5.05	5.6 vs. 5.7	5.75 vs. 5	5.85 vs. 5	5.75 vs. 5.85
	p values	p = 0.0005	p = 0.0012	n.s. p = 0.9949	p = 0.0007	p = 0.0007	n.s. p = 0.9910

Significant differences (p < 0.05) were analyzed by applying the log-rank (Mantel-Cox) test. n = 6 mice/group. n.s.: not significant differences.

Other groups of C57BL/6 mice immunized as described in Table 1 were challenged 2 weeks after the last immunization by administering an i.v. injection of 2000 spz of *Pb-Pv*CSP247. Percent protection to reach T1% and parasitemia are shown in Fig. 5 (e,f and g,h, respectively). Mice immunized with formulations containing the Montanide adjuvant presented a significant delay in the T1% period compared to the adjuvant-only groups (Fig. 5f and Table 2, p = 0.0005). Additionally, a significant delay in the T1% period (p = 0.0049) was observed in mice immunized with NLP-CSP_CT_ protein formulated with the Poly (I:C) adjuvant (Fig. 5e and Table 2).

Finally, a third set of C57BL/6 mice immunized as described in Table 1 were challenged 2 weeks after the last immunization by administering an i.v. injection of 2,000 spz *Pb-Pv*CSP-like G10. Percent protection to reach T1% and parasitemia are shown in Fig. 5 (i, j and k, l, respectively). With either adjuvant, a statistically significant delay in the T1% period in mice immunized with either protein compared to the adjuvant-only groups was observed (p = 0.0007 for Montanide adjuvant-treated groups and p < 0.0015 for Poly (I:C) adjuvant-treated groups, Table 2). As in previous experiments using *Pb-Pv*CSP210 and *Pb-Pv*CSP247 parasites, no significant differences were observed between the naïve (not immunized, infected mice) and adjuvant-only groups (data not shown).

## Discussion

The development of an effective vaccine would be an important tool against malaria, as it would provide a cost-effective form of prevention and would help circumvent adaptive strategies both from the vector and parasite.

In addition to the general obstacles to overcome when developing vaccines against parasitic diseases, research groups developing vaccines against *P. vivax* face other issues*.* One of them is the formation of hypnozoites, which can cause relapses within months and even years after the primary infection^49^. Our vaccine formulations were able to elicit high and long-lasting titers of *Pv*CSP-specific antibodies in mice, providing partial protection against challenge with chimeric *P. berghei* spz expressing the *P. vivax* CSP variants. Since a significant proportion of sporozoites released in the challenge were prevented from causing an infection, our formulation could hypothetically contribute to the reduction in cases of relapse, as it would prevent the formation of new hypnozoites in the liver^49^.

Another important obstacle to overcome is that *P. vivax* does not infect rodents. For this reason, the preclinical evaluation of the protective efficacy of vaccine formulations is mainly restricted to the use of monkeys. In addition to the ethical conflict associated with the use of NHP in such early stage of the vaccine development, these animals must undergo a splenectomy to facilitate the development of parasitemia. This procedure may not provide robust data since organ removal causes immunological changes^50^. Thus, a strategy that enables the preclinical determination of protective immune responses induced by vaccine formulations against *P. vivax* malaria is based on the use of chimeric parasites expressing *P. vivax* proteins that are the targets of the vaccine formulations^51^. In particular, the use of transgenic parasites in the study of CSP-based vaccine formulations for the pre-erythrocytic phase of infection has allowed the analysis of functional inhibition of the exogenous CSP, expressed in replacement of the endogenous protein. Following this strategy, chimeric *P. berghei* parasites expressing the *P. vivax* CSP variants VK210 and VK247 were used to determine the protective efficacy of vaccine formulations consisting of viral vectors carrying *P. vivax* CSP alleles^25,41–43^. In this study, we generated for the first time a chimeric *P. berghei* parasite line expressing the third *Pv*CSP variant, *Pv*CSP-*P. vivax*-like.

We first attempted to develop a chimeric parasite in which the endogenous *P. berghei* csp gene was replaced with the *P. vivax*-like csp gene. Unfortunately, this transgenic parasite failed to produce visible spz inside oocysts, and only very few in the salivary glands of *An. stephensi* mosquitoes. This failure to complement the function of the endogenous CSP was also observed in transgenic *P. falciparum* expressing *P. vivax* CSP as replacement lines^44^. In addition, the chimeric *P. berghei Pb-Pv*CSP247 line produces significantly less salivary gland spz than the *Pb-Pv*CSP210 line^25^. To overcome this concern, we generated an “additional copy” chimeric parasite in which the endogenous *P. berghei* csp gene is maintained and the *P. vivax-like* csp gene is expressed under the control of the promoter region of the sporozoite- and liver-specific *P. berghei* gene *uis4.* This strategy was successfully applied in previous studies, in which chimeric *P. berghei* spz have been generated by introducing a *P. falciparum csp* gene as an additional copy into the *P. berghei* genome. These chimeric *P. berghei* spz expressed both *Pb*CSP and *Pf*CSP at their surface^45–47^. Additionally, chimeric *P. falciparum* spz have been generated by introducing a *P. vivax csp* gene as an additional copy into the *P. falciparum* genome. These chimeric *P. falciparum* spz also expressed both *Pv*CSP and *Pf*CSP at their surface^52^, similarly to our results.

The development of the first chimeric *P. berghei* parasite expressing the *Pv*CSP-*P. vivax*-like protein allows the preclinical determination of protective immunity of vaccines targeting this mostly neglected CSP variant. The genome of *P. vivax*-like as a malaria-transmitting parasite in apes has been recently published^53^. Similar to *P. knowlesi* and *P. simium* infections in humans, *P. vivax*-like malaria could currently be considered a zoonotic disease, probably with continuing cross-species exchange of *P. vivax* between humans and apes in tropical Africa^16^. This hypothesis is based on not only the shared vector species (*An. vinckei*, *An. moucheti*, and *An. marshallii*) but also their low host specificity and high longevity^54^. Nevertheless, a significant proportion of this variant was found in *P. vivax* infections of patients from endemic areas of the Brazilian Amazon, as determined by molecular methods^17–19^. Consistent with these findings, Soares et al. (2020) recently reported the high (~ 40–60%) prevalence of antibodies against *P. vivax*-like in patients from three communities in this region^55^. These results prompted us to propose that human-to-human transmission is very likely. The aim of this work is not to elucidate whether human-infective *P. vivax*-like is a *P. vivax* allelic variant or a different species causing zoonosis; however, regardless of the origin and classification, we cannot continue to neglect actions to combat it. Therefore, in this work, we developed the first chimeric *P. berghei* parasite expressing the *Pv*CSP-*P. vivax*-like protein, and used these parasites to analyze protective immunity in mice immunized with recombinant proteins representing all three *P. vivax* CSP variants.

Our recombinant proteins include the repeats of the *Pv*CSP VK210 and VK247 variants and a sequence representing *P. vivax-*like CSP. Thus, our formulations would be predicted to be effective against a broad spectrum of cases of vivax malaria, caused not only by *P. vivax*, but *P. simium* and *P. vivax*-like as well. This could represent an improvement when compared to formulations such as VMP001^56^ and Rv21^25^, which contain only sequences of *Pv*CSP VK210 and VK247, as would confer higher protection against *P. vivax*-like infections, than that expected to be achieved through cross-reactivity from other CSP allelic variants. Moreover, our immunization data show that our vaccine formulations stimulated the production of high titers of specific antibodies against each of the variants. In previous studies, it was demonstrated the absence of significative cross-reaction or antigenic interference among the *Pv*CSP-repeat sequences in animals immunized with individual (VK210, VK247 and *P. vivax*-like) recombinant proteins, produced in bacteria^57^ or yeast^22^. Besides it, formulations containing the chimeric fusion protein, comprising epitopes of all three different allelic forms, were as immunogenic as the mixture of three individual *Pv*CSP proteins. Thus, the specific response to *Pv*CSP RI, repeats and C-terminal regions combined in our formulations might contribute substantially to enhancing protective efficacy, since specific antibodies against these regions are highly neutralizing^41^, thus indicating the importance of a universal formulation.

Although our vaccine formulations did not confer sterile protection after challenge with chimeric spz, the significant delay in T1% periods was noteworthy for several reasons, as described below.

i. Each day of delay in blood-stage parasitemia is representative of ~ tenfold fewer sporozoites reaching the liver^58^, and as previously discussed, this decrease would impact hypnozoite formation, thus preventing relapses^49^. ii. Due to technical reasons, we used our previously established i.v. challenge system, which does not allow us to consider the effect of specific CSP antibodies that potentially act in the skin. In the case of *Plasmodium* infections, these types of antibodies were recently shown to contribute significantly to protective effects^59,60^. iii. In natural infections, most mosquitoes inoculate only ~ 1% of the sporozoites in their salivary glands, with median inocula ranging between ~ 40 and 100 sporozoites^61^. Supporting this, it was demonstrated that a natural infection with 8 *P. berghei*-infected mosquitoes is equivalent to i.v. inoculation of 250–500 sporozoites^60^. Therefore, our challenge system (i.v. inoculation of 2000 spz) is stronger than other strategies using intradermal or s.c. challenge. The variation in the challenge system also explains the apparent discrepancies in protective efficacy comparing our results with previous studies^23^ (30% mice protected in the s.c. challenge system vs. significant delay in T1% in the i.v. system). In agreement, a similar situation was observed comparing the protective performance of previous *Pv*CSP-based formulations using s.c.^22^ and i.v.^43^ challenges, respectively. Taking into account all these facts, the effectiveness of the formulations developed in this study might be even greater when considered in the case of natural infection.

Consistent with a previous study^43^, a clear positive relation was observed between high titers of CSP-specific antibodies and protection. In a comparison of both recombinant proteins, the delay in the T1% period obtained with NLP-CSP_CT_ was longer than with NLP-CSP_R_ when administered with the same adjuvant. Most likely, antibodies against the C-terminal domain of *Pv*CSP, which is absent in NLP-CSP_R_, are responsible for the differences, as this region might be important for protection^41^. The lack of anti-C-terminal antibodies and the lower titers against VK247 repeats in vaccines using Poly (I:C) as an adjuvant reported in previous work^23^ would explain the lack of protection provided by NLP-CSP_R_/Poly (I:C) against challenge with *Pb-Pv*CSP247 parasites. By the other hand, the C-terminal region of *Pv*CSP contains predicted T cell epitopes, which could also contribute to protective effect of pre-erythrocytic vaccines^40^. However, in previous studies, very low levels of *Pv*CSP-specific CD4^+^ and CD8^+^ T cell responses were elicited by our *Pv*CSP recombinant proteins^22,57^. Results from the VMP001 clinical trial also indicate the low contribution of repeats and C-terminal region to induce T cell responses, as only 17% of vaccinated subjects responded to these antigens whereas 90% showed strong cellular responses to the N-terminal region^56^ (absent in NLP-CSP proteins). Moreover, it was shown that PvCSP short repeat-region peptides, when presented on a VLP, can induce antibodies mediated protection^41^. Nonetheless, we do not exclude the participation of T cell-mediated immune responses in the protection observed in this study.

Finally, Montanide ISA720 was overall a better adjuvant in terms of IgG titers and protective efficacy. However, phase I clinical trials showed some concerns regarding the safety of Montanide ISA720-adjuvanted vaccines against malaria, particularly high reactogenicity^62–64^.

For all these reasons, we aim to analyze the mechanisms underlying the observed protection before moving into clinical testing of safety and toxicology. Ongoing research analyzing the specificity of humoral and cellular responses and performing transcriptomic analysis of lymphocytes from immunized mice will provide insights into the pathways that are selectively activated by these formulations and will provide valuable information about the type of immune response that a protective vaccine against vivax malaria should elicit.

## Supplementary information

Supplementary Information.
