# Supplementary material for: A universal vaccine candidate against Plasmodium vivax malaria confers protective immunity against the three PvCSP alleles
Source: Sci Rep. 2021 Sep 9;11:17928. doi: 10.1038/s41598-021-96986-1 (PMC8429696; doi:10.1038/s41598-021-96986-1)
Supplement: Supplementary file 1 — Supplementary Information. [file 41598_2021_96986_MOESM1_ESM.docx]

**A universal** **vaccine candidate against *Plasmodium vivax* malaria confers protective immunity against the three *Pv*CSP alleles**

**- Supplementary Material -**

Alba Marina Gimenez^1,2,§^, Ahmed M. Salman^1,§^, Rodolfo F. Marques^2^, César López-Camacho^1^, Kate Harrison^1^, Young Chan Kim^1^, Chris J. Janse^3^, Irene S. Soares^2*^ and Arturo Reyes-Sandoval^1,4*^

^1^Nuffield Department of Medicine, The Jenner Institute, University of Oxford, The Henry Wellcome Building for Molecular Physiology, Roosevelt Drive, Oxford, OX3 7BN, UK.

^2^Department of Clinical and Toxicological Analyses, School of Pharmaceutical Sciences, University of São Paulo, São Paulo, SP, Brazil.

^3^Department of Parasitology, Leiden Malaria Research Group, Center of Infectious Diseases, Leiden University Medical Center, (LUMC, L4-Q), Albinusdreef 2, ZA Leiden, 2333, The Netherlands.

^4^Instituto Politécnico Nacional, IPN. Av. Luis Enrique Erro s/n. Unidad Adolfo López Mateos, Zacatenco. CP 07738. Mexico City. México.

^§^These authors should be considered joint first authors.

*Corresponding authors

# Table S1. Primers for genotyping the chimeric *P. berghei* line *Pb-Pv*CSP-like G10 (2700 cl1)

| **Primer No.** | **Descirption** | **Primer sequences** |
| --- | --- | --- |
| **1048** | **hDHFR-yFCU (+/-SM) F** | ATCATGCAAGACTTTGAAAGTGAC |
| **1049** | **hDHFR-yFCU (+/-SM) R** | CATCGATTCACCAGCTCTGAC |
| **1051** | **PvCSP-Like G10@Pbuis4 5'Int. F** | GTCGCCAGTCAAGTAACAAC |
| **1080** | **PvCSP-Like G10@Pbuis4 5'Int. R** | ACTGTTATATTTGGTGATGGAATGG |
| **1081** | **PvCSP-Like G10@Pbuis4 3'Int. F** | TATACATCCACGGATGCATAGAAG |
| **1083** | **PvCSP-Like G10@Pbuis4 3'Int. R** | TTCAGTGAAATCGCAAACATAAGTATC |
| **1302** | **PvCSP-Like G10_F** | ATGAAGAACTTCATTCTCTTGGC |
| **1303** | **PvCSP-Like G10_R** | AGTTAATTGAATAATGCTAGGAC |

# Table S2. Oocyst and sporozoite production of *Pb-Pv*CSP-like G10 (2700 cl1) and *Pb-Pv*G10(r) (2710 cl1) and wild type (WT) *P. berghei* ANKA (676m1 cl1)

| **Parasite line** | **Line number** | **Oocyst no.  Mean ± SD** | **Sporozoites no.  Mean** | **Prepatent period^1^ days** |
| --- | --- | --- | --- | --- |
| **WT *P. berghei*** | 676m1 cl1 | 216 ± 22 | 18,800 | 5.8 |
| ***Pb-Pv*CSP-like G10** | 2700 cl1 | 132 ± 27 | 4,600 | 6.1 |
| ***Pb-Pv*G10(r)** | 2710 cl1 | 108 ± 24 | <200 | N.D. |

N.D: not determined
^1^: Prepatent period: the time to 1% parasitaemia after intravenous injection of 1000 sporozoites in BALB/c mice


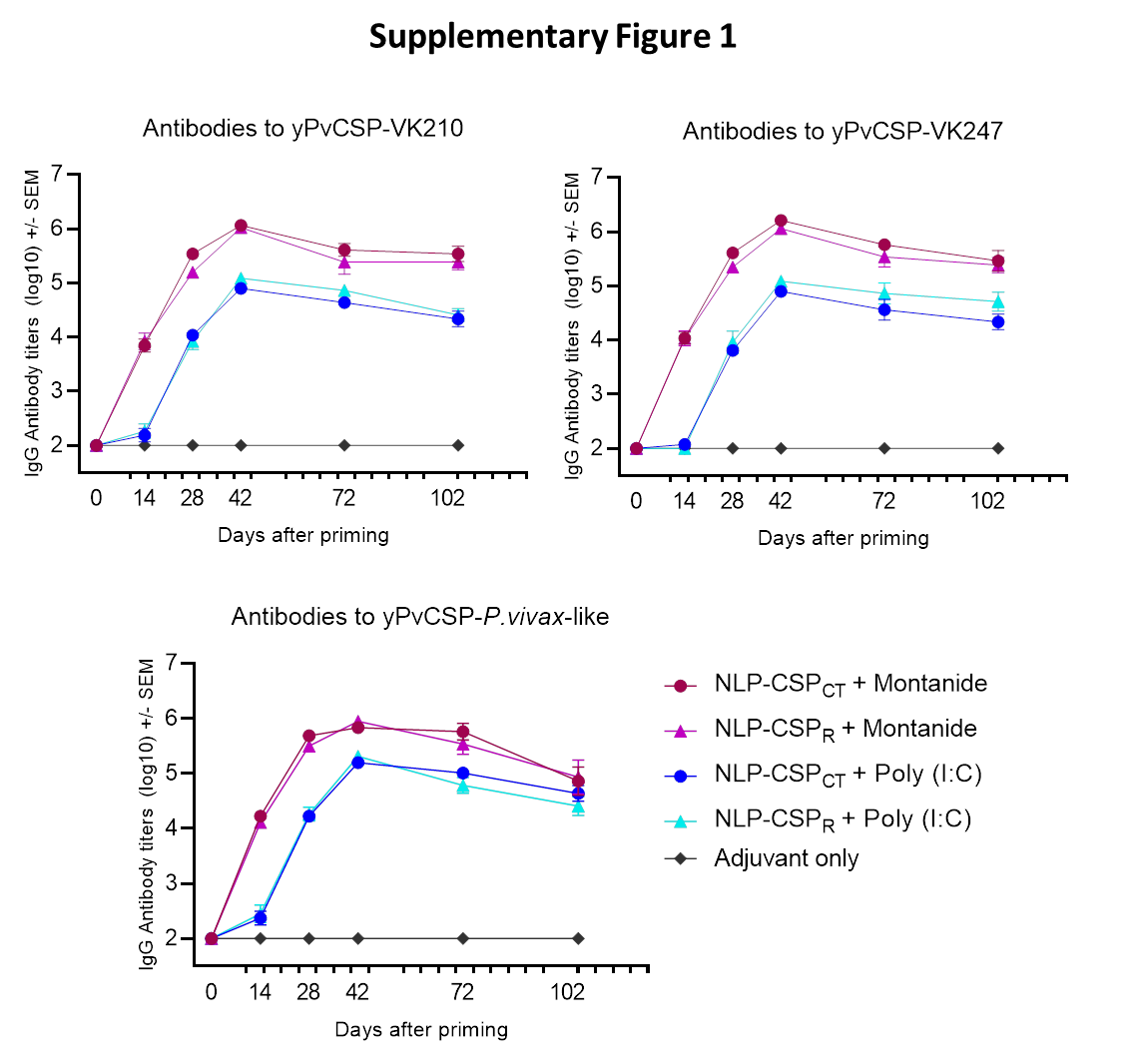


**Supplementary Figure 1**. **Longevity of IgG antibodies** **in mice immunized with recombinant proteins.**

C57BL/6 mice were s.c. immunized with the recombinant proteins NLP-CSP_CT_ and NLP-CSP_R_ in the presence of Poly (I:C) or Montanide ISA 720 adjuvants using the scheme shown in Table 1. IgG antibody titers were determined using ELISA assays at days 0, 14, 28, 42, 72 and 102 after priming. The individual *Pv*CS proteins (y*Pv*CSP-VK210, y*Pv*CSP-VK247 and y*Pv*CSP-*P. vivax*-like) were used as solid-phase bound antigens.


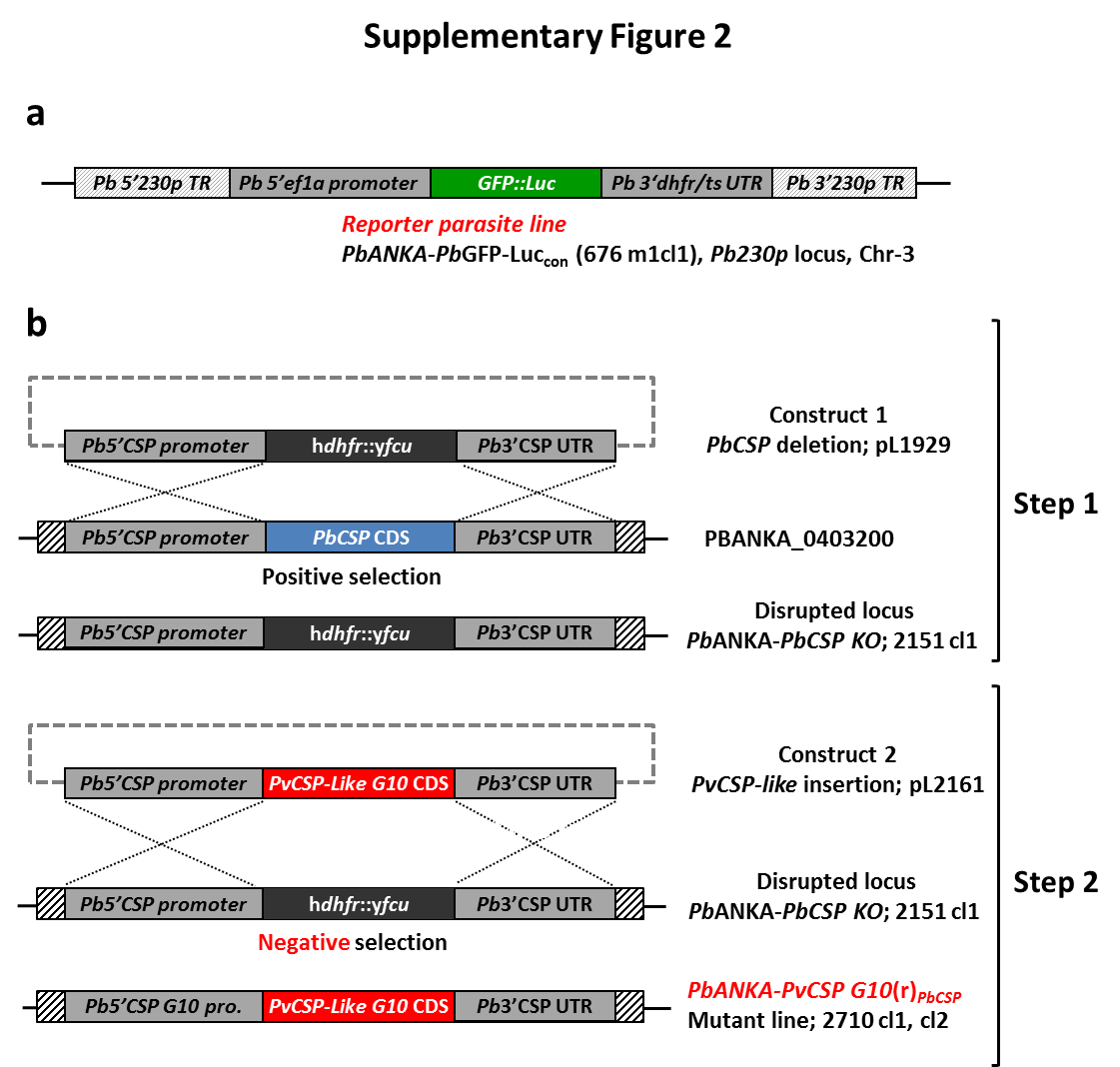


**Supplementary Figure 2. Strategy to generate chimeric** ***P. berghei* parasite line expressing a *PvCSP-P. vivax-*like protein.** A replacement line where the *P. berghei csp* coding sequence is replaced with the *Pv*CSP-*P. vivax*-like CDS (PVU09738) was generated using a 2-step GIMO transfection protocol.

**a**. Schematic representation of the transgenic *p230p* locus of the reporter *Pb*ANKA parasite line *PbGFP-Luc_eef1α_* (676m1cl1), which was used to generate the single replacement gene [SRG] chimeric parasite line (see b)*.* This reporter line expresses a GFP and firefly luciferase (LUC-IAV) fusion protein under the constitutive *Pbeef1a* promoter and is selectable marker (SM)-free. The reporter cassette is integrated into the neutral *p230p* locus on chromosome 3.

**b**. Schematic representation of the generation of the chimeric line *Pb-Pv*G10(r) (2710cl1 and 2710cl2) where in step 1, the GIMO deletion construct (construct 1; pL1929) is used to replace the *Pbcsp* coding sequence (CDS) on chromosome 4 with the positive/negative selectable marker (SM; h*dhfr::*y*fcu*) cassette, resulting in the generation of the *Pbcsp* GIMO line (*Pb*ANKA-*Pb*CSP GIMO; line 2151 cl1) after positive selection with pyrimethamine. Construct 1 targets the *Pbcsp* gene by double crossover homologous recombination. In step 2, the GIMO insertion construct (construct 2; pL2161) is used to replace the SM in the *Pbcsp* GIMO line with the *Pv*CSP-*P. vivax*-like CDS after negative selection using 5-fluorocytosine (5-FC), generating the transgenic line *Pb-Pv*G10(r) (2710 cl1, 2710cl2). Construct 2 integrates the transgene by double crossover homologous recombination using the same targeting regions (TRs) employed in construct 1, resulting in the introduction of the *Pv*CSP-*P. vivax*-like CDS under the control of the *Pbcsp* gene promoter and transcriptional terminator sequences and removal of the SM.


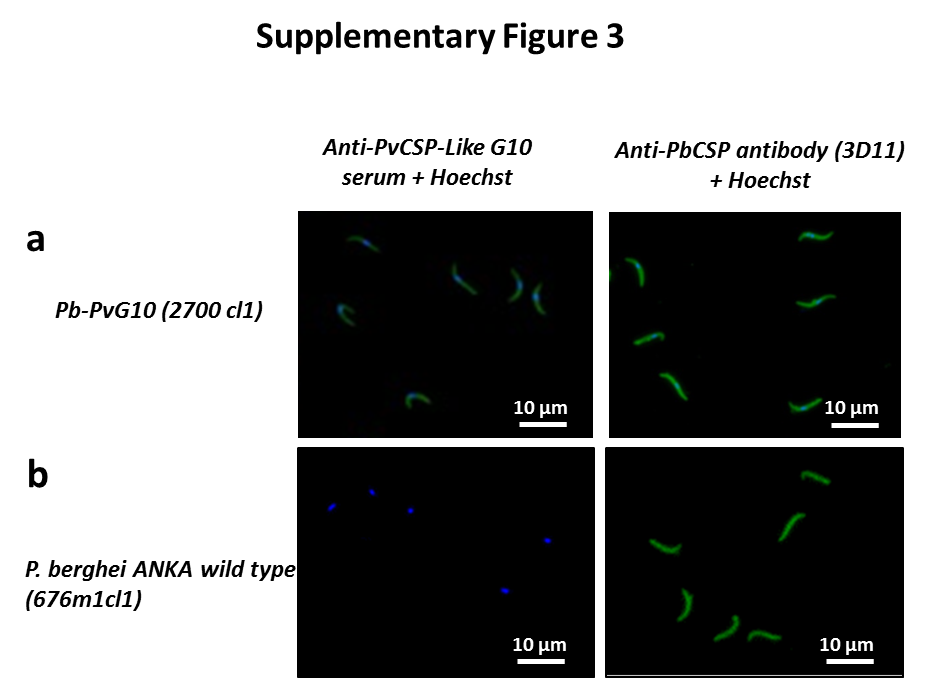


**Supplementary Figure 3. Phenotype analyses of the chimeric *Pb-Pv*CSP-like G10 line (2700 cl1) expressing *Pv*CSP-*P. vivax*-like protein under control of the *Pbuis4* promoter.**

Expression of the *Pv*CSP-*P. vivax*-like protein in spz was analysed by performing an immunofluorescence assay using sera from mice immunized with the recombinant proteins. As a control, the 3D11 antibody recognizing *P. berghei* CSP was used. **a.** Immunofluorescence analysis showing the expression of both *Pv*CSP-*P. vivax*-like and *Pb*CSP in sporozoites of *Pb-Pv*CSP-like G10. (Alexa Fluor 488, green; nuclear staining with Hoechst 33342). **b.** As a control, wild-type (676m1cl1) *P. berghei* sporozoites were stained with the same sera and monoclonal antibody. Merged images of the different channels are shown for both chimeric and wild-type *P. berghei* sporozoites.


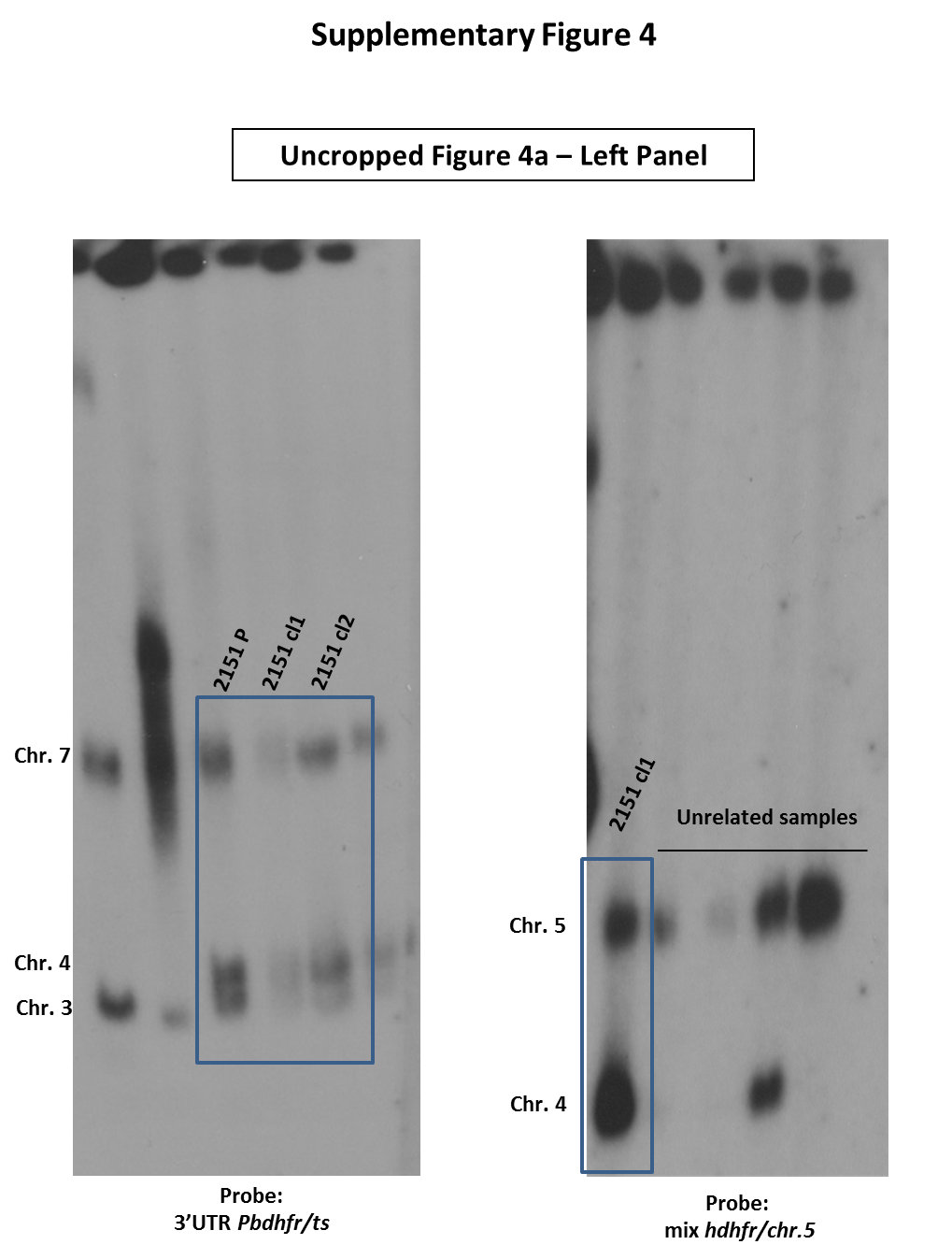


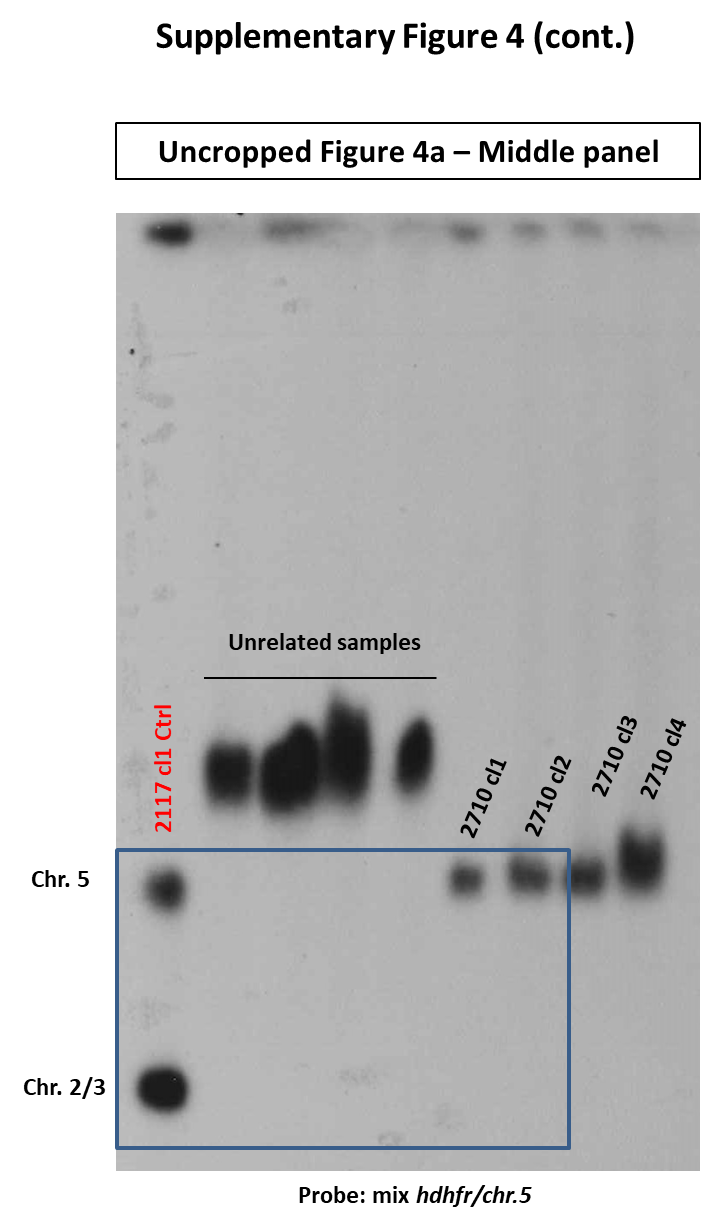


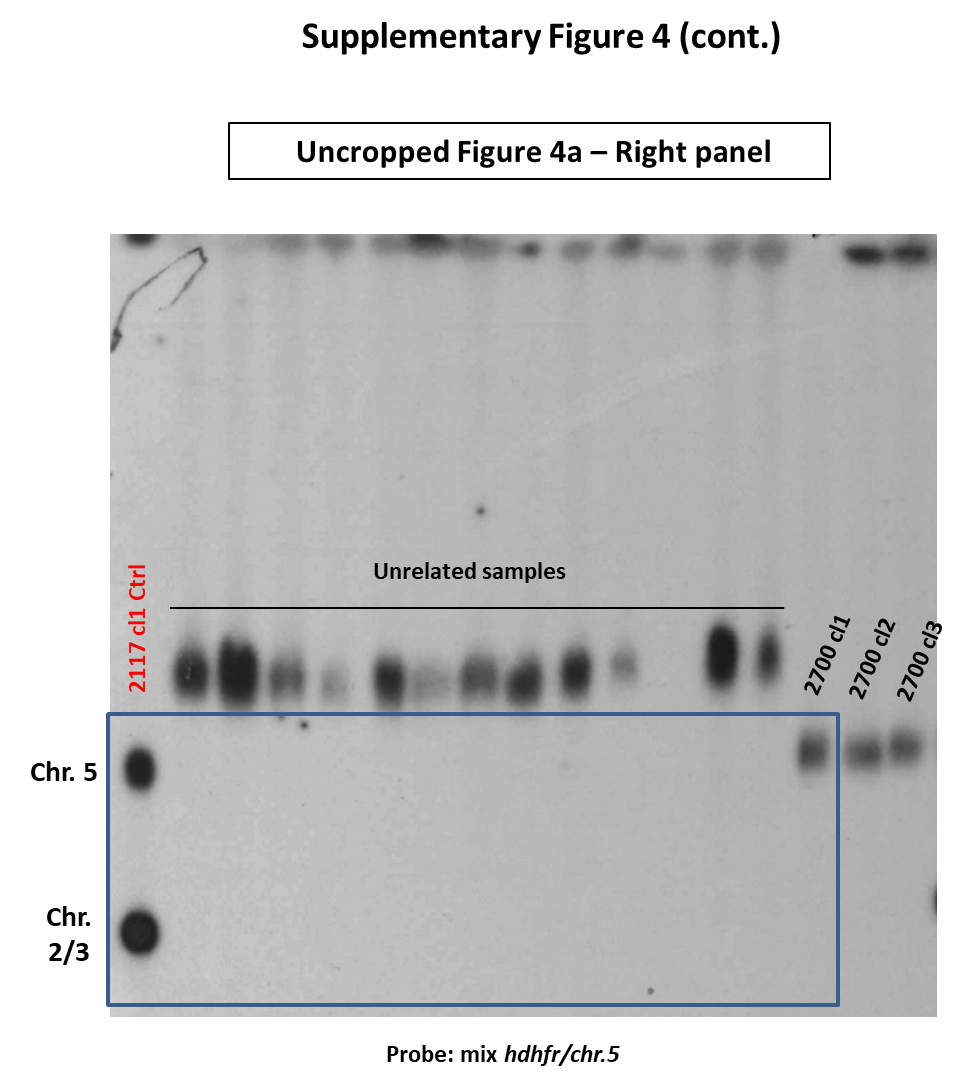


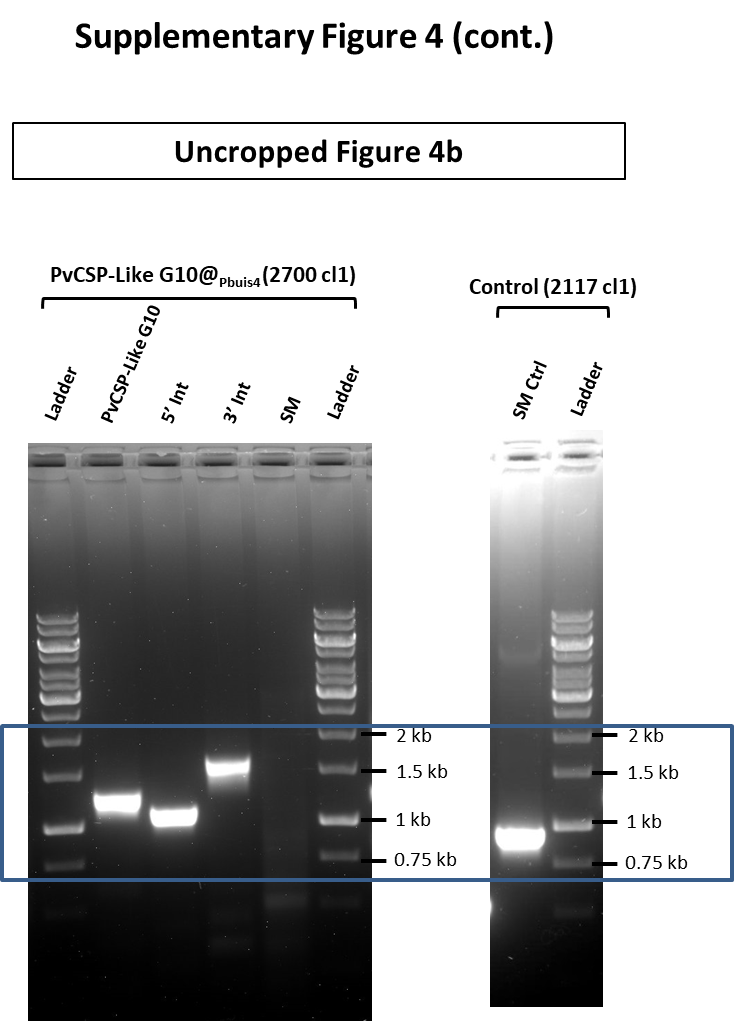


**Supplementary Figure 4. Full-length blots/gels presented in Figure 4.** Boxed regions correspond to cropped images presented in figure 4.
